# FDG uptake heterogeneity in FIGO IIb cervical carcinoma does not predict pelvic lymph node involvement

**DOI:** 10.1186/1748-717X-8-294

**Published:** 2013-12-23

**Authors:** Frank J Brooks, Perry W Grigsby

**Affiliations:** 1 Department of Radiation Oncology, Washington University School of Medicine, 4921 Parkview Place, Saint Louis, MO 63110, USA; 2 Division of Nuclear Medicine, Mallinckrodt Institute of Radiology, Medical Center, Saint Louis, MO, USA; 3 Department of Obstetrics and Gynecology, Washington University Medical Center, Saint Louis, MO, USA; 4 Alvin J Siteman Cancer Center, Washington University Medical Center, Saint Louis, MO, USA

**Keywords:** Cervical cancer, FDG-PET, Intra-tumor heterogeneity, Image texture analysis

## Abstract

**Translational relevance:**

Many types of cancer are located and assessed via positron emission tomography (PET) using the 18F-fluorodeoxyglucose (FDG) radiotracer of glucose uptake. There is rapidly increasing interest in exploiting the intra-tumor heterogeneity observed in these FDG-PET images as an indicator of disease outcome. If this image heterogeneity is of genuine prognostic value, then it either correlates to known prognostic factors, such as tumor stage, or it indicates some as yet unknown tumor quality. Therefore, the first step in demonstrating the clinical usefulness of image heterogeneity is to explore the dependence of image heterogeneity metrics upon established prognostic indicators and other clinically interesting factors. If it is shown that image heterogeneity is merely a surrogate for other important tumor properties or variations in patient populations, then the theoretical value of quantified biological heterogeneity may not yet translate into the clinic given current imaging technology.

**Purpose:**

We explore the relation between pelvic lymph node status at diagnosis and the visually evident uptake heterogeneity often observed in 18F-fluorodeoxyglucose positron emission tomography (FDG-PET) images of cervical carcinomas.

**Experimental design:**

We retrospectively studied the FDG-PET images of 47 node negative and 38 node positive patients, each having FIGO stage IIb tumors with squamous cell histology. Imaged tumors were segmented using 40% of the maximum tumor uptake as the tumor-defining threshold and then converted into sets of three-dimensional coordinates. We employed the sphericity, extent, Shannon entropy (*S*) and the accrued deviation from smoothest gradients (*ζ*) as image heterogeneity metrics. We analyze these metrics within tumor volume strata via: the Kolmogorov-Smirnov test, principal component analysis and contingency tables.

**Results:**

We found no statistically significant difference between the positive and negative lymph node groups for any one metric or plausible combinations thereof. Additionally, we observed that *S* is strongly dependent upon tumor volume and that *ζ* moderately correlates with mean FDG uptake.

**Conclusions:**

FDG uptake heterogeneity did not indicate patients with differing prognoses. Apparent heterogeneity differences between clinical groups may be an artifact arising from either the dependence of some image metrics upon other factors such as tumor volume or upon the underlying variations in the patient populations compared.

## Introduction

There is evidence that pelvic lymph node status is an important indicator of both disease free and overall survival in patients diagnosed with cervical carcinoma [1,2]. There is also a growing belief that the uptake heterogeneity observed within tumors assayed via FDG-PET may be of prognostic value [3,4]. A correlation between increased FDG uptake heterogeneity and worse prognosis has been reported for human sarcomas [5,6], non-small cell lung cancer [7], esophageal cancer [8] and cervical cancer [9,10]. Although the relationship is not well-understood, it seems that tumor state (e.g., quiescence or rapid proliferation) likely is strongly linked to tumor metabolism [11]. Because FDG uptake is some measure of metabolic activity within the tumor, this connection to tumor state offers one plausible means for uptake heterogeneity to indicate disease outcome. We therefore hypothesize that increased intra-tumor FDG uptake heterogeneity may correlate with lymph node involvement. If such a link were established, it will provide a new avenue of studying why only some of cervical carcinoma patients exhibit pelvic lymph node involvement and perhaps even provide insight as to why that status so profoundly affects prognosis.

**Figure 1:**
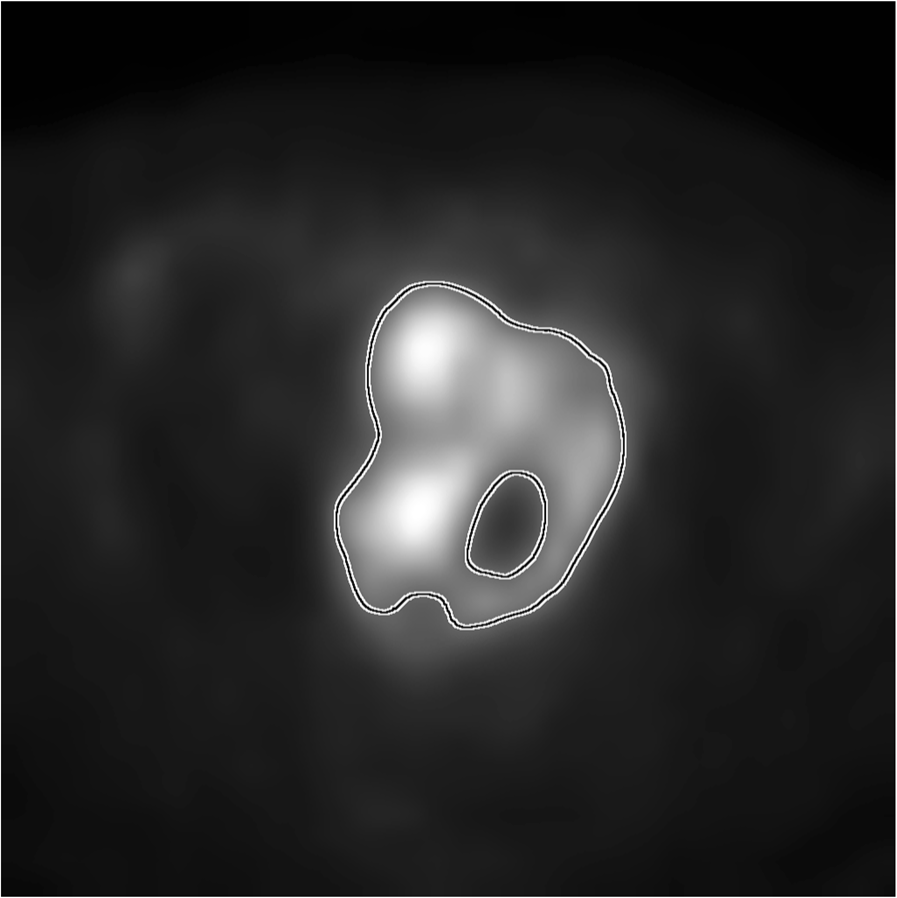
**Shown is an example of a cervical carcinoma imaged via FDG-PET.** The black and white contour approximately indicates the defined tumor boundary. Within, there are clear variations in the grayscale intensity. It is these variations researchers seek to objectively quantify such that inter-patient comparisons of *intra*-tumor uptake heterogeneity can be made. The image edges correspond to 20 cm within the patient.

Heterogeneity in FDG uptake is observed in FDG-PET images as variations in grayscale intensity with more intense regions ostensibly corresponding to increased metabolic activity. Examples may be seen in Figure 1 where variations in grayscale intensity are clearly visible. Of course, each image is a low-resolution view of the underlying biology and is heavily attenuated by the partial volume effect [12]. Therefore, the image heterogeneity observed is unlikely to directly correspond to the tumor biology from whence the detected signal came [4,13]. For this reason, all FDG uptake heterogeneity analyses should be taken *cum grano salis* when attempting to establish or infer biological meaning from the analyses.

The image heterogeneity observed may be thought of as comprising variations in intensity: distribution, arrangement and shape. The grayscale intensity distribution results from some combination of underlying biology, scanner noise and the partial volume effect. These are the shades available to create image detail; less shades means that less variation can be conveyed. The spatial arrangement of the intensities also conveys variation. For example, intuitively, the smooth gradation of the brightest image intensities to the dimmest is less varied than those same intensities randomly juxtaposed. Overall shape is distinct from arrangement because shape defines *a priori* the bounds of where the intensities may be arranged. For example, a tumor growing near a physical barrier may be unable to achieve the same shape a tumor protruding into a void might achieve. These considerations are crucial when determining if two distinct intensity samples (*i.e.*, tumor regions) differ significantly.

There have been several previous attempts at quantifying uptake heterogeneity seen in FDG-PET images [5-10,14-16]. While we do not exhaustively review them here, we recapitulate some apposite concerns. One study analyzed almost twenty higher-order texture metrics and found only a few to be statistically reproducible on FDG-PET images [17]. This is consistent with another study which showed that dozens of texture metrics applied to FDG-PET images of cervical carcinomas were not useful indicators of prognosis [10]. In general, texture metrics often are inter-related and thus including additional metrics might not include additional information. For example, the image entropy is (qualitatively) inversely proportional to the image energy [18]. In practice, similar dependences between other metrics may be may not be at all intuitive. For example, it was argued that the slope of the SUV-volume histogram was an intra-tumor heterogeneity metric with prognostic value [9], however, later that same metric was shown to be a surrogate for tumor volume [19]. An analogous dependence upon tumor volume also was illustrated with virtual tumor images for the local image entropy [20]. Still another metric, the area under the *cumulative* SUV-volume histogram, also was presented as a prognostic heterogeneity measure [7,14]. It was argued later that that metric is equivocal in the sense that it is possible for unequal heterogeneity scenarios to achieve identical metric values while unequal metric values can result from identical heterogeneity scenarios [21,22]. Quantification of image heterogeneity is a difficult problem in general and has only recently been addressed in the specific context of predicting disease outcome from FDG-PET images. Therefore, challenges and refinements to previously proposed metrics (including those we employ) are to be expected as the mathematical sophistication of the problem is revealed via further study.

We analyze our image data via a spatial metric which has been demonstrated to be both independent of tumor volume and consistent with visual ranking of FDG-PET images by human experts [16]. While this metric does provide some measure of the variation in intensity arrangement within pre-established tumor regions, it also is sensitive the overall region shape [16]. We employ the sphericity and extent as distinct, volume-independent shape metrics which may provide complementary information about the contribution of shape to perceived heterogeneity. The sphericity and extent are metrics which can distinguish rounder, more compact regions from highly asymmetrical, porous regions [23]. We employ the well-established Shannon entropy [18,24] as the measure of informational content within the individual distributions of grayscale intensities. We note however, that the Shannon entropy is predicted to depend non-linearly upon tumor volume and that the exact functional form of that dependence is influenced by both the intensity histogram bin size and image segmentation threshold employed [20].

## Materials and methods

### Patients

This is a retrospective study of 85 patients with newly diagnosed cervical cancer who underwent FDG-PET or FDG-PET/CT at Washington University in St. Louis between July 2003 and March 2012. This study was approved by the Washington University Human Research Protection Office. All patients were evaluated by history and physical examination, examination under anesthesia, and FDG-PET or integrated PET/CT before initiating treatment. Patients were staged clinically, according to FIGO staging (AJCC 2002, 6th edition). The selection criteria for inclusion into this study were FIGO clinical stage IIb tumors and squamous cell histology. The patients ranged in age from 27 y to 85 y with a median of 49 y. The maximum SUV of the tumors analyzed ranged from 4 to 56 and approximately followed a log-normal distribution with median of 13 and variance of 74.

### Identification of primary tumors

For patients (*n* = 58) examined between July 2003 and June 2008 diagnostic imaging scanning was performed via Siemens Biograph 2 (Munich, Germany) while for those examined (*n* = 27) between October 2008 and March 2012 were scanned via Siemens Biograph 40 (Munich, Germany). In both cases, images were reconstructed via ordered subset expectation maximization with 8 sets; 2 iterations for the former and 4 iterations for the latter. In both cases, a Gaussian smoothing filter was applied post-reconstruction with 5.3 mm and 4 mm full width at half maximum, respectively. We note that the 9 most recent Biograph 40 image sets underwent an additional point-spread function/time-of-flight correction. The primary tumor evident in each FDG-PET image set was identified and segmented specifically for the present study by a veteran oncologist using MIM version 5.6.3 (MIM Software Inc., Cleveland, OH). Within the manually approximated tumor region, any image voxel with SUV greater than 40% of the maximum region SUV was considered to be part of the tumor [25]. The oncologist then made slight manual adjustments to the ROI to remove any obvious non-tumor pixels such as those comprising bladder or bowel regions. For each patient, these data were exported as a set of (*x,y,z*) coordinates, each with a single 15-bit grayscale image intensity corresponding to radioactivity density in Bq/mL.

### Assessment of FDG uptake heterogeneity

We assessed observed FDG uptake heterogeneity in several ways. First, we compute the Shannon informational entropy (S) upon the histogram of grayscale intensities for each FDG-PET image set [18,24]. The histogram bin width was defined for each patient individually as the Freedman-Diaconis bin width. We used the formula

(1) $$-\overset{B}{\underset{b=1}{\sum }}p_{b}\ln\;p_{b}$$

where *pb* is the probability that a given grayscale intensity resides in the *b*th of a total of *B* histogram bins. From the (*x,y,z*) coordinates, we computed the sphericity (*ψ*) and the extent (*ξ*) for each virtual tumor object. The sphericity is defined as [23,26]

(2) $$\frac{\sqrt[{3}]{\pi }{\left(6\cdot \text{volume}\right)}^{\frac{2}{3}}}{\text{surface}\;\text{area}}$$

The extent is defined as the ratio of the net object area (or volume) to a bounding area (or volume) [23]. We note one difference from the usual definition of the extent. Instead of a bounding box, we employed a bounding sphere with diameter matching the maximal diameter of the tumor object and centered at the geometric center of the tumor object. For example, a largely hollow but basically round tumor will have a low extent (i.e., few pixels in the bounding sphere) but a high sphericity. Thus, *ψ* and *ξ* together form a set of complementary metrics which together feasibly discern heterogeneous from homogeneous tumor geometry. Lastly, we compute the accrued deviation from smoothest gradients (*ζ*) [16]. Automated ranking of tumor image via increasing *ζ* has been shown to be consistent with rankings done visually by human experts. As described in Ref. [16], we computed *ζ* for our three-dimensional intensity data by using tri-linear interpolation to approximate the 15-bit intensity value whenever a fractional (*x,y,z*) coordinate was required (recall that only integer coordinates are stored within single FDG-PET image).

### Statistical analysis

Potential differences between the distribution of a heterogeneity statistic for distinct patient subgroups defined by pelvic lymph node status were assessed via the two-sample Kolmogorov-Smirnov test. This test of empirical cumulative distribution functions (CDFs) was chosen because it: does not require modeling of the distributions compared, can compare continuous data sets of unequal size and/or variance and is sensitive to more than just differences in distribution location [27]. The subgroups compared are those with no indication of pelvic lymph node involvement (LN-) to those with (LN+). For our population comprising 47 LN- and 38 LN+ patients, a maximum difference (*D*) between the CDFs greater than 0.286 is significant at the 95% level [28]. In cases where cross-group ties amongst ranks occur, the *p*-value given for D is only an upper-bound of the true *p*-value [27]. Specific cases of additional data stratification with differing significance criteria are detailed in the Results section, as necessary. Where appropriate, we employ Kendall’s *τ* as a measure of variable association and test other potential variable dependencies via *χ*^2^-test applied to contingency tables. In cases where histograms were constructed, the Freedman-Diaconis bin width was employed. All statistical analyses were performed using R version 2.15.2 (R Foundation for Statistical Computing, Vienna, Austria).

## Results

**Figure 2:**
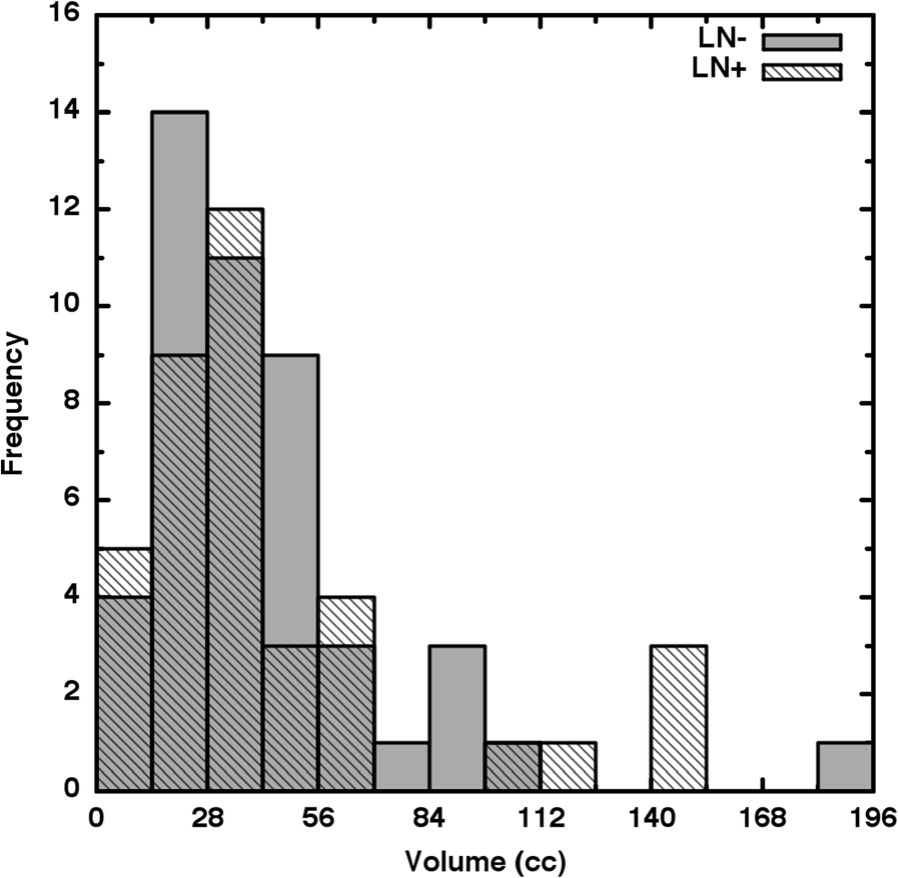
**The common-axis histograms overlap largely and thus show that volume alone cannot predict to which lymph node status groups a patient will belong.** In other words, we find no appreciable difference in distribution of volumes between the LN- and LN+ patient groups. Histogram bin size = 14 cc.

**Figure 3:**
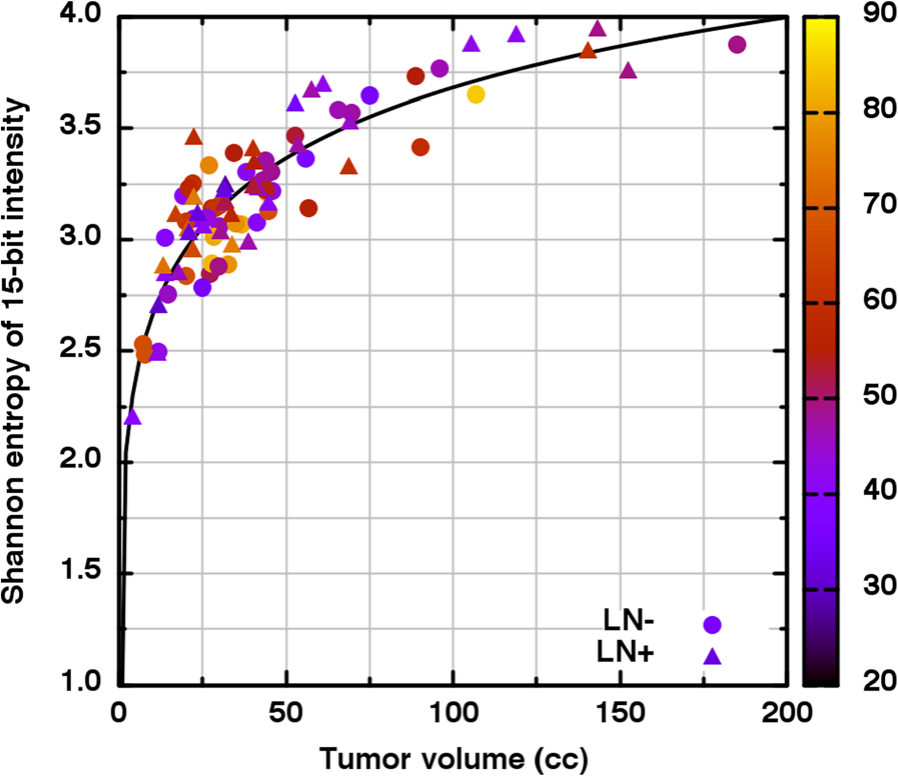
**The Shannon informational entropy ( *S *) of each 15-bit grayscale intensity histogram is seen to depend monotonically upon tumor volume ( *V *) across a wide range of volumes.** It is thus seen that by using increasing *S* values to rank tumors in increasing heterogeneity, one actually is ranking those tumors coarsely in terms of volume. The age of the patients in years is indicated by color.

In Figure 2, the histograms for the LN- (shaded) and the LN+ (hatched) groups are shown on the same axes. The Kolmogorov-Smirnov test (*D* = 0.121; *p* ≤ 0.918) implies no significant difference between these tumor volume distributions. In Figure 3, *S* is seen to be strongly dependent upon tumor volume (*V*). Thus, direct comparison of S for large volumes to *S* at small volumes is not appropriate. We therefore stratified *V* into quartile groups of similarly sized tumors and compared the *S* distributions of the lymph node status subgroups strictly within each volume stratum. Table 1 shows the results where it is seen that *S* does not differ significantly within any volume stratum. We repeated this analysis for *ψ* and *ξ* separately and again found no significant difference for either variable in any volume stratum (Table 1).

**Table 1:** Within each volume stratum, the Kolmogorov-Smirnov test was used to discern potential differences between lymph node status groups for each of the heterogeneity metrics

**Volume (in cc)**	**Shannon entropy ( *S *)**	**Sphericity ( *ψ *)**	**Spherical extent ( *ξ *)**
4.0 ≤ V < 22.4	*D* = 0.227; *p =* 0.886	*D* = 0.355; *p =* 0.390	*D* = 0.200; *p* ≤ 0.985
22.4 ≤ V < 32.8	*D* = 0.548; *p* ≤ 0.102	*D* = 0.471; *p* ≤ 0.222	*D* = 0.269; *p* ≤ 0.865
32.8 ≤ V < 52.8	*D* = 0.173; *p =* 0.987	*D* = 0.500; *p* ≤ 0.168	*D* = 0.539; *p =* 0.078
52.8 ≤ V < 185.1	*D* = 0.364; *p =* 0.479	*D* = 0.182; *p* ≤ 0.993	*D* = 0.273; *p* ≤ 0.808

The relatively high p-values implies that none of the metrics differ significantly across lymph node status groups.

**Figure 4:**
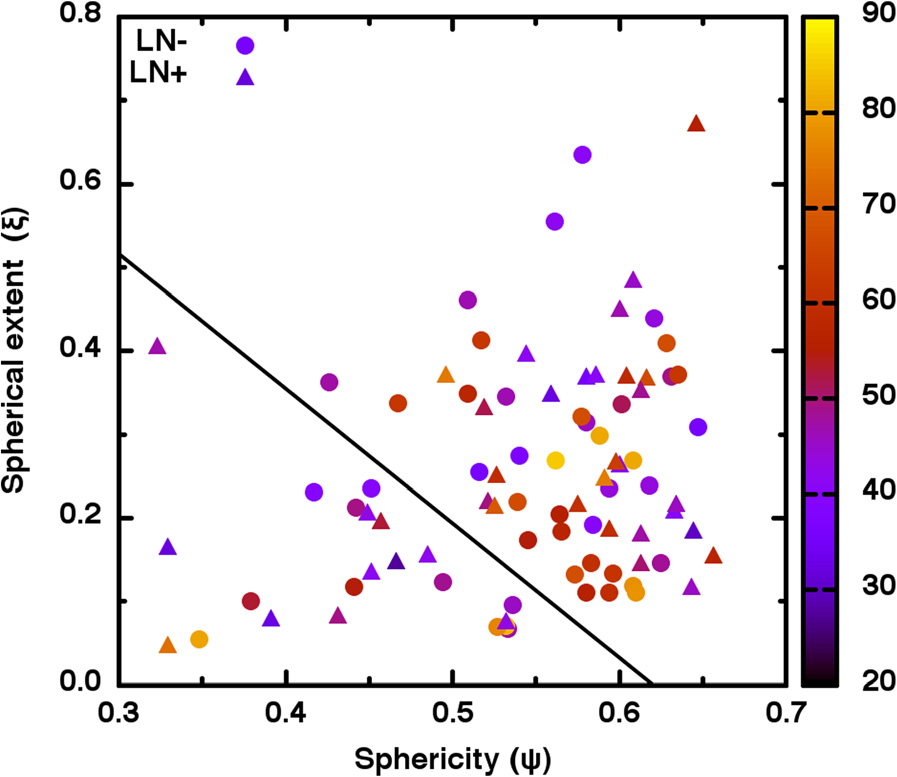
**Although the combination of shape metric with percent shape-filling metric feasibly might identify heterogeneous tumors, a contingency table analysis indicated otherwise.** As is seen, no clear clustering of LN- or LN+ patients occurs despite the appearance of a clear gap (indicated by the solid line) in shape coordinates. The age of the patients in years is indicated by color.

To analyze the potential discerning power of *ψ* and *ξ* together, we first plot (*ψ*, *ξ*) coordinate pairs. As seen in Figure 4, there are no obvious clusters of LN- (circles) and LN+ (triangles) and the variables *ψ*, *ξ* are only weakly correlated (*τ* = 0.164). With some subjectivity, one might see a gap in coordinate location indicated by the solid line in Figure 4. However, a contingency table analysis of lymph node status versus position relative to that line yields *χ*^2^ = 0.149, *p =* 0.699. This indicates that we may not reject the null hypothesis that lymph node group membership is independent of (*ψ*, *ξ*) coordinate location. In other words, no clearly defined region of (*ψ*, *ξ*) coordinate space reliably corresponds to a particular lymph node group.

**Figure 5:**
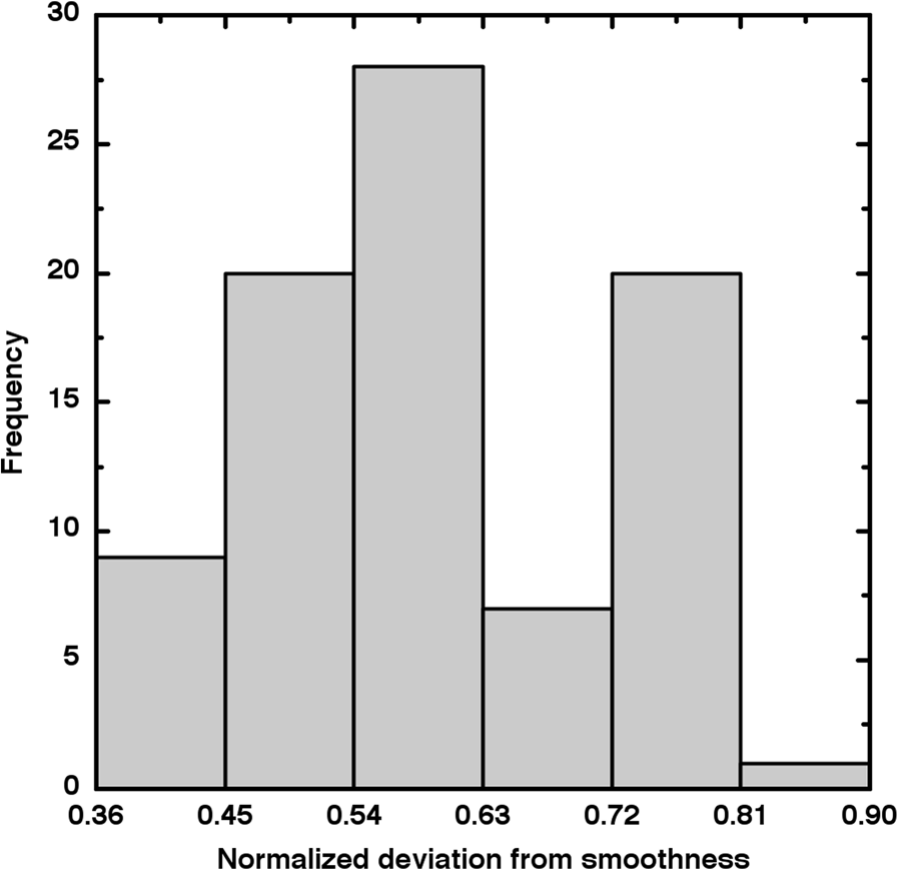
**The distribution of *ζ***_***n***_**values appears to be bimodal, thus implying a possible threshold between “small” and “large” *ζ***_***n***_**.** However, a contingency table analysis does not support a correspondence between lymph node status and this heterogeneity metric. Histogram bin size = 0.09.

We note that *ζ* is only weakly correlated with previous shape (*τ* = -0.271 for *ζ*-*ψ*; *τ* = -0.275 for *ζ*-*ξ*) and intensity heterogeneity metrics (*τ* = 0.063 for *ζ*-*S*) and therefore plausibly might distinguish heterogeneity groups in ways those metrics could not. We applied the Kolmogorov-Smirnov test to *ζ* and again found no significant difference between the different lymph node groups (*D*=0.147; *p* ≤ 0.752). We did, however, observe that *ζ* is moderately correlated (*τ* = 0.610) to the mean grayscale intensity (*g*). We investigated this relation in two ways. First, we define a new variable *ρ* as the first principal component of the matrix of *ζ*-*g* correlations. We found that *ρ* represented 89% of the total *ζ*-*g* variance. Proceeding as before, we found (*D* = 0.215, *p =* 0.239) which indicates no significant difference in *ρ* between lymph node groups. Second, we modified the *ζ* described in Ref. [16] by dividing the average deviation from smoothest gradients by the maximal deviation found for each patient *before* computing the area the under the curve. This yields a new variable 0 ≤ *ζ*_*n*_ ≤1 which still measures deviations from homogeneity but is only weakly dependent upon *g* (*τ* = -0.142). Although the Kolmogorov-Smirnov test again indicated no difference between lymph node subgroups (*D* = 0.148; *p* ≤ 0.744), a natural break in *ζ*_*n*_ was observed. In Figure 5, distinct modes appear across the gap in *ζ*_*n*_ values between 0.63 and 0.72. We therefore segment the global patient group into small and large heterogeneity groups at *ζ*_*n*_ = 0.675. A *χ*^2^-test conducted on that 2×2 contingency table (*χ*^2^ = 0.172, *p =* 0.678) indicates that lymph node status likely does not depend upon *ζ*_*n*_.

## Discussion

We now note some important aspects of our uptake heterogeneity analysis. First, our patient population was chosen in order to reduce variation in characteristics which affect FDG-PET image heterogeneity. For example, one might expect image differences between squamous and non-squamous histology precisely because of the differences in tissue type and/or density. Additionally, because FIGO stage is known to have prognostic value, but the relation between stage and FDG-PET uptake is not well understood, stage could affect the image data in unknown ways. Our patients are all of the same FIGO stage and histology. This increases the believability that the statistical distribution of a given uptake heterogeneity metric comes from intra-tumor variability and not from comparison of unlike tumors.

Second, we specifically consider of the relation between uptake heterogeneity metrics and tumor volume. The plot of informational entropy versus volume given in Figure 3 is but one ready example of how heterogeneity metrics should be expected to depend upon tumor volume [13,20]. Consider that the tumor volume is nothing more than the size of the sample chosen from the distribution of FDG-PET intensities possible for a given tumor type. Decreasing the sample size (volume) must decrease the believability that the underlying distribution has been represented represented and therefore must also decrease the reliability of comparisons to much larger samples. In our analysis, we employed metrics proven to be volume independent or, in the case of the informational entropy, specifically stratified our patient data as to diminish the dependence upon volume. We may therefore be more certain that any observed uptake differences are not the result of the comparison of disparate volumes.

Third, we analyze the original FDG-PET image data unaltered via changes in image bit depth. This is crucial because any heterogeneity metric, by definition, must be sensitive to the image bit depth. Consider that visually evident differences in tumor heterogeneity might be described verbally as differences in image: detail, variation, texture, tone, contrast and/or grain. By reducing the number of grayscale shades available to render those qualities, the entire image is smoothed. That is, reducing bit depth *always* decreases heterogeneity. However, vast cross-patient differences in tumor shape, tumor size, orientation (relative to the scanner) and maximum FDG uptake imply that a bit depth reduction can affect each data set differently. Thus, while reducing *image* variation for individuals, population variation actually could be enhanced by bit depth reduction. For example, consider a case where two differently shaped and shaded tumors happen to yield similar heterogeneity metric values. Because bit depth reduction affects shade more than shape, one tumor can be smoothed significantly more than the other, thus yielding tumors with different metric values in the new bit depth. This is another way that the statistical distribution of uptake heterogeneity metric values can depend upon patient population more strongly than upon intra-image variation (which is what ostensibly is being measured).

Our main result is that none of the previously established uptake heterogeneity metrics we employed yielded statistically significant differences between patients with pelvic lymph node involvement and those without. In other words, we found FDG uptake heterogeneity does not predict membership to groups with differing prognoses. This is counter to previous claims that measurements of intra-tumor uptake heterogeneity generally are of prognostic value [5-10,14]. We posit two main reasons those analyses yielded contradictory results. First, the precise uptake heterogeneity found in studies of other tumor types (such as, e.g., non-squamous small cell lung carcinomas) simply may not be applicable to, or even possible in, the cervical carcinomas we study. Second, and more generally, is the lack of variation in our patient population. In brief, when patient populations vary in ways which plausibly affect FDG-PET image heterogeneity metrics, differing heterogeneity was found; when this population variation was controlled, differing heterogeneity was not found. Indeed, it is *possible* that all apparent prognostic FDG uptake heterogeneity is a statistical effect arising from the partial volume attenuation of vastly differing tumor types, shapes and sizes.

To objectively isolate tumor from background, we employed the 40% of maximum threshold established for cervical carcinomas [25]. This threshold is conservative in that it almost certainly misses some tumor at the periphery. The point of using a conservative threshold is so that one may be reasonably assured that the data analyzed actually correspond to *intra*-tumor regions. As the tumor-defining threshold is lowered, so too is the believability that the desired intra-tumor measurements aren’t skewed by inclusion of background pixels. If, instead, the threshold were raised significantly, it is unlikely that heterogeneity even could be measured when so few pixels of so few shades are given as input. In essence, a super-conservative threshold virtually guarantees homogeneity.

Another reasonable concern about our analysis is in our use of image data from two scanners. The sphericity and extent were computed using Euclidean distances in centimeters. While is true that the older scanner cannot give as precise an estimate of size as does the current one, it should not give an inaccurate estimate. Both the conversion of radioactivity to grayscale intensity and the histogram binning of those intensities were uniquely determined for each patient. The deviation from smoothest gradients is derived on a percent of size, percent of intensity scale *a priori*. For these reasons we expect that use of multiple scanners is not a significant source of data heterogeneity in our analysis.

One weakness of our analysis in the creation of the *ζ*_*n*_ statistic. The original, unbounded heterogeneity metric *ζ*[16] only weakly correlates with the normalized metric *ζ*_*n*_ (*τ* = 0.040). Therefore while *ζ* is a metric proven to correlate with heterogeneity rankings done visually by human experts, *ζ*_*n*_ is unproven. Although it is reasonable that *ζ*_*n*_ can distinguish image sets, the exact interpretation of *ζ*_*n*_ or its correspondence with particular visually perceived image properties is not established. We may therefore only say that a feasible measure of image variation (*ζ*_*n*_) did not distinguish patient groups of differing lymph node status and that this lack of distinction is consistent with the results using proven metrics (*S* and *ζ*). Another potential weakness is in the use of the sphericity (*ψ*) and extent (*ξ*) to assess heterogeneity in tumor shape. In general, *ψ* measures the closeness to spherical shape and *ξ* measures the percent filling of that shape. Because neither situation is likely to occur for real tumors, use of *ψ* and *ξ* imposes an unrealistic shape constraint. This means that less variation is possible *a priori* and genuine differences in tumor shape are compressed into a smaller range of variable value. Also, these and similar metrics can exhibit non-linear jumps in value. When segmenting an image, some thresholding process is employed to discern tumor from background. This means, for example, that a ring-like cross-sectional shape could be opened to a horseshoe-like shape simply due to slight signal variations which do not surmount the intensity threshold. The result is that biologically similar tumors could suddenly become mathematically different when assessed by metrics strongly dependent upon the ratio of interior pixels to surface pixels. We visually inspected every image of our entire data set and found holes and/or disconnected tumor regions to occur in 218 of the 1,328 images analyzed. Given the strength of our other results, we do not feel that this particular source of error will change our overall conclusions, however, this is one more example of how the analysis of FDG-PET images is inherently more challenging than the commonality of the metrics employed belie.

## Conclusion

We analyzed a patient population homogeneous in qualities which plausibly affect the image heterogeneity observed in FDG-PET assays of some cervical carcinomas. We found that established metrics of image heterogeneity do not indicate involvement of pelvic lymph nodes. Because pelvic lymph node involvement is a demonstrated prognostic indicator, our results seem at odds with studies which suggest intra-tumor FDG uptake heterogeneity has prognostic value. We argue that the potential conflict might be explained by the heterogenous nature of the patient populations in other studies influencing the statistical distribution of some image heterogeneity metrics.

## Competing interests

Frank Brooks and Perry Grigsby have no conflicts of interest with the publication of this manuscript.

## Authors’ contribution

FJB drafted the manuscript and performed all mathematical analyses. PWG designed the protocol for the interpretation the FDG-PET images, acquired the image and lymph node status data presented, and provided crucial medical insight into the data analyzed. Both FJB and PWG read and approved the final manuscript.
